# Three Dimensional Imaging of Paraffin Embedded Human Lung Tissue Samples by Micro-Computed Tomography

**DOI:** 10.1371/journal.pone.0126230

**Published:** 2015-06-01

**Authors:** Anna E. Scott, Dragos M. Vasilescu, Katherine A. D. Seal, Samuel D. Keyes, Mark N. Mavrogordato, James C. Hogg, Ian Sinclair, Jane A. Warner, Tillie-Louise Hackett, Peter M. Lackie

**Affiliations:** 1 μ-VIS Centre, Faculty of Engineering and the Environment, University of Southampton, Southampton, United Kingdom; 2 James Hogg Research Centre, University of British Columbia, Vancouver, Canada; 3 Sir Henry Wellcome Laboratories, Faculty of Medicine, University of Southampton, Southampton General Hospital, Southampton, United Kingdom; 4 Department of Anesthesiology, Pharmacology and Therapeutics, University of British Columbia, Vancouver, Canada; University of Southern California, UNITED STATES

## Abstract

**Background:**

Understanding the three-dimensional (3-D) micro-architecture of lung tissue can provide insights into the pathology of lung disease. Micro computed tomography (µCT) has previously been used to elucidate lung 3D histology and morphometry in fixed samples that have been stained with contrast agents or air inflated and dried. However, non-destructive microstructural 3D imaging of formalin-fixed paraffin embedded (FFPE) tissues would facilitate retrospective analysis of extensive tissue archives of lung FFPE lung samples with linked clinical data.

**Methods:**

FFPE human lung tissue samples (n = 4) were scanned using a Nikon metrology µCT scanner. Semi-automatic techniques were used to segment the 3D structure of airways and blood vessels. Airspace size (mean linear intercept, Lm) was measured on µCT images and on matched histological sections from the same FFPE samples imaged by light microscopy to validate µCT imaging.

**Results:**

The µCT imaging protocol provided contrast between tissue and paraffin in FFPE samples (15mm x 7mm). Resolution (voxel size 6.7 µm) in the reconstructed images was sufficient for semi-automatic image segmentation of airways and blood vessels as well as quantitative airspace analysis. The scans were also used to scout for regions of interest, enabling time-efficient preparation of conventional histological sections. The Lm measurements from µCT images were not significantly different to those from matched histological sections.

**Conclusion:**

We demonstrated how non-destructive imaging of routinely prepared FFPE samples by laboratory µCT can be used to visualize and assess the 3D morphology of the lung including by morphometric analysis.

## Introduction

The airways, vasculature and lymphatics of the human lung form a complex interrelated three dimensional (3D) network that structurally ranges from macroscopic to microscopic scales. Macroscopic structures greater than 2.3 mm in diameter can be visualized *in vivo* using clinical multi-detector computed tomography (MDCT) scanners [1]. For diseases such as asthma and chronic obstructive pulmonary disease (COPD) the small airways with internal diameters of less than 2mm with no cartilage support [2,3] are thought to be the major site of airflow obstruction. Thus clinical MDCT scanners do not have the resolution to resolve small airways (>2 mm in diameter) affected by disease. In order to understand the microstructure of the lung a higher magnification and improved resolution is needed. In particular 3D reconstruction of tissue features allows us to understand structural changes and changes in the spatial relationship of the tissue components in a way that is otherwise impossible [4]. Using serial sections is very labor intensive, requiring significant expertise in sample preparation, imaging and image reconstruction. Micro CT (μCT) scanners provide an alternative with resolutions of up to 1 μm per voxel [5–7]. The use of μCT has previously been to image air-inflated and fixed whole murine lung [8] or human lung samples [3,9] visualising small airways and alveolar structures without physically sectioning the sample. Further, it has been shown that stereological methods can be applied to such μCT images to quantify lung morphometry (i.e. luminal dimensions, acinar volume, volume fractions and surface area) [10]. Thus μCT has a significant advantage over traditional histology because it offers non-destructive 3D imaging of the micro-structure in a given sample. Specifically volumetric μCT imaging can therefore be used to localize structures of interest within a given tissue sample and can facilitate the diagnosis and staging of disease. In addition scouting for regions of interest within a larger tissue volume has potential to decrease the blinded, laborious and costly sectioning of entire samples and improve the representative sampling of potentially heterogeneous tissues.

To enable studies of lung disease severity many investigators have established archival biobanks that contain tissue samples representative of multiple disease states (for example the UBC James Hogg Research Centre (JHRC) Biobank [11] containing in excess of 50,000 samples). Traditionally, histological methods for fixation and storage of tissues have focused on formalin-fixation, for the conservation of tissues and paraffin infiltration and embedding to ensure consistent sectioning with a microtome. Paraffin has a similar density to tissue which has precluded μCT analysis of formalin-fixed paraffin-embedded (FFPE) samples due to the low contrast between tissue and paraffin. Paraffin has a density of 0.9 g/mL at 20°C [12] whereas the normal (“healthy”) lung tissue has a density between 0.32 g/mL at functional residual capacity (FRC) and 0.13 g/mL at total lung capacity (TLC) [13,14] as measured by CT. These differences are substantially reduced by the effective infiltration of tissue with paraffin during embedding. It is generally recognized that soft tissue imaging is a challenge for CT given the modest tissue attenuation levels. Therefore to maximize the contrast between the tissue and the surrounding medium, the preparation of lung tissue samples within the literature has involved the use of contrast agents or air-inflation prior to fixation. Thus, the purpose of this study was to develop a protocol that enables non-destructive imaging of routinely prepared or archival FFPE samples by μCT, to visualize the 3D morphology of the lung. Parts of this study have been previously published in abstract form [15,16].

## Materials and Methods

### Human Lung Tissue Samples

Surgically resected human lung tissue was obtained with written informed consent from two patients undergoing elective surgery at University Hospital Southampton, UK and two patients at St Paul’s Hospital, Vancouver, Canada. A copy of the consent is kept with the study files as approved as part of the studies by the research ethics board of each institution. Southampton ethics was from the National Research Ethics Service Committee, South Central—Southampton A, number 08/H0502/32, Vancouver ethics was from University of British Columbia-Providence Health Care Research Institute Research Ethics Board, number H05-01150. In Southampton tissue samples of ~1.5 cm^3^ were fixed in neutral buffered formalin for 48 hours in tissue cassettes and embedded in paraffin wax following a standard protocol using a Shandon Hypercenter tissue processor (Fisher Scientific, Loughborough, UK). For the Vancouver samples lungs were first inflated with formalin and then sampled as previously described using a cylindrical cutting tool [17]. The cylindrical tissue cores of 15 mm by 7 mm were then infiltrated with low melting point paraffin, using a Leica tissue processor.

### μ-CT imaging protocol

**Fig 1:**
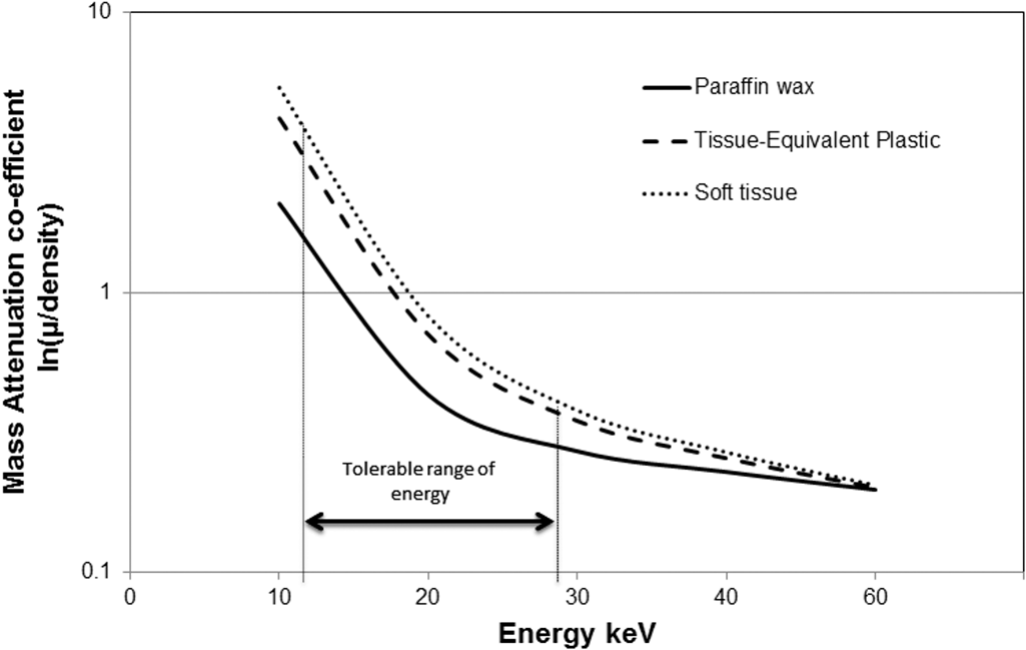
Mass attenuation coefficients versus X-ray energy for paraffin wax and soft-tissue. The tolerable range indicates an acceptable difference in mass attenuation between the paraffin and tissue, for samples thicknesses down to approximately 5mm.

The FFPE lung samples were scanned using a custom-built Nikon Metrology μCT scanner, with a 225kV X-ray source, flat panel detector with a high sensitivity CsI scintillator, and carbon fibre entrance window for improved low energy performance (Perkin-Elmer 1621, 2048 x 2048 pixel). An electron accelerating voltage of 50kV was used, with a molybdenum reflection target, yielding a mean energy in the order of 18KeV, along with characteristics peaks around 17 and 19keV. No filtration was used. A beam current of ~150μA was selected, yielding a total beam energy below 8W, avoiding damage to the target and the corresponding need to defocus the spot (i.e. increasing geometrical unsharpness in the image). Fig 1 illustrates the mass attenuation coefficients of soft tissue and the paraffin wax based on standard National Institute of Standards and Technology (NIST) data [18,19]. As such it may be seen that whilst the present imaging conditions reduce available flux (in terms of accelerating voltage and target atomic number), they access the attenuation contrast between tissue and standard paraffin mounting medium (*i*.*e*. below ~40keV). 3D reconstructions with voxel resolutions between ~6 and 12μm were created, dependant on sample size, using standard filtered-back projection (Ram-Lak filter) within the Nikon CT Pro 2.0 package using 3142 projections (360° rotation in 0.11° increments). Sample shuttling was used to remove ring artefacts, requiring a stop-start rotation mode, with typical exposure times of 1s and 8 to 16 frame averaging per projection, giving total scan durations in the order of 12 hours.

### Image visualization and analysis

To explore the lung sample data, the 3D visualization and analysis package VGStudio Max (Volume Graphics, Heidelberg, Germany) was used to select representative sub-volumes and to generate volume rendered images. Semi-automatic techniques were used to segment airways and blood vessels in the lung tissue. Briefly, an approximate region of interest was defined around a vessel or airway and an automatic 3D seed growth tool (VGStudio Max) was then used to delineate connected structures. A user defined seed point in the structure was used and connected voxels with grey values matching within a defined tolerance to the seed were added to the segmented volume.

Additionally, the Fiji distribution of ImageJ (version 1.48p), an image processing and analysis software package [20] was used for semi-automatic airway tracings and analysis of airspace size (mean linear intercept [21]) in the micro CT images of the FFPE lung samples. The mean linear intercept values, measured by two expert readers (DMV, TLH), were obtained from 10 μCT images and 10 matched serial histological sections to provide validation of such measurements at the image resolution provided by μCT. The data were analyzed using Bland-Altman plots to determine intra-observer variability and the differences between image modalities.

### Image scouting and histology

Following μCT scanning FFPE samples were set in paraffin blocks for sectioning. After sectioning, sections were de-paraffinized and stained using Movat’s pentachrome stain [22]. The sections were then imaged using a 10x objective on a Dot-Slide scanning system (Olympus, Southend-on-Sea, UK). Multiple images were then montaged to form an image of the whole section. The matching image plane in the μCT image stack was then identified and extracted using VG-Studio, by matching 3 or more structural features in the sample. To allow direct comparison an ImageJ plugin (UnwarpJ elastic registration [23]) was then used to align the histological and μCT images to each other after initially matching three or more structural features in the sample.

## Results

**Fig 2:**
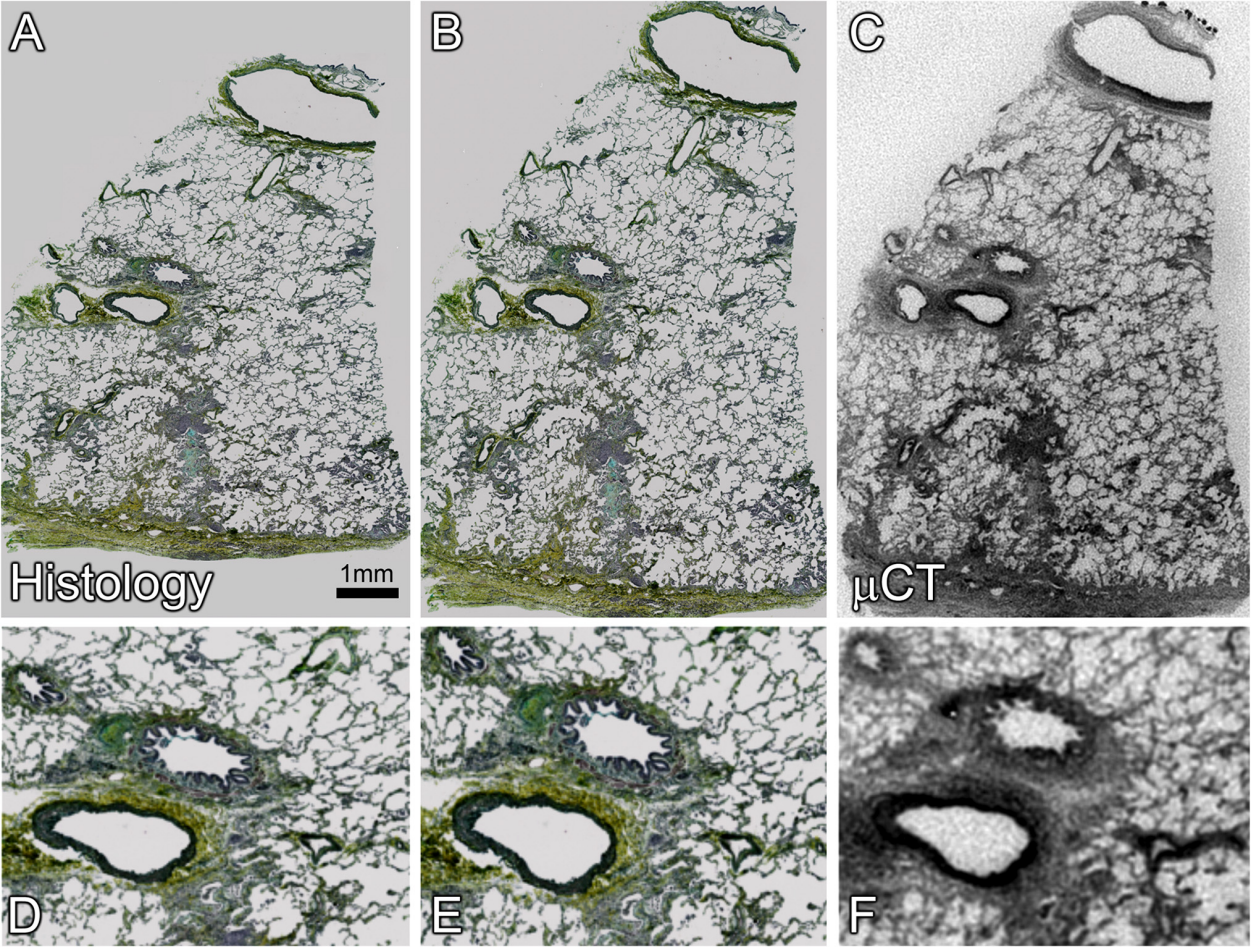
Registration based image matching of μCT and histological section stained with Movat’s Pentachrome. A. shows the histological section imaged using a 10x objective on a Dot-Slide scanning system. The initial image required deformation, rotation and translation using UnwarpJ elastic registration [23] to match the μCT image, reflecting deformation during sectioning. The final transformed image of the histological section (B) and with a matching slice from the corresponding μCT image (C). Details of the same sub-region in A, B and C are shown in D, E and F. Structural details in the μCT image correspond closely to those seen by light microscopy.

Here we demonstrate that the μCT imaging protocol described in the methods enables the necessary contrast between tissue and paraffin for visualization of microstructures within FFPE samples. Specifically for FFPE samples of 15 mm x 7 mm in size the reconstructed images have a voxel size of 6.9 μm. As demonstrated by Fig 2, with this μCT imaging protocol airways and blood vessels are distinguishable on the basis of their wall composition and branching pattern. The resolution provided by the scanner also allows the delineation of the fine microstructure of the alveolar airspaces. To ensure that the images obtained by μCT were consistent with light microscopy, the FFPE samples following scanning were sectioned for histology. Fig 2 shows an example of the image matching between sections stained with Movat’s pentachrome and the corresponding μCT section. Image registration was performed to align the images.

**Fig 3:**
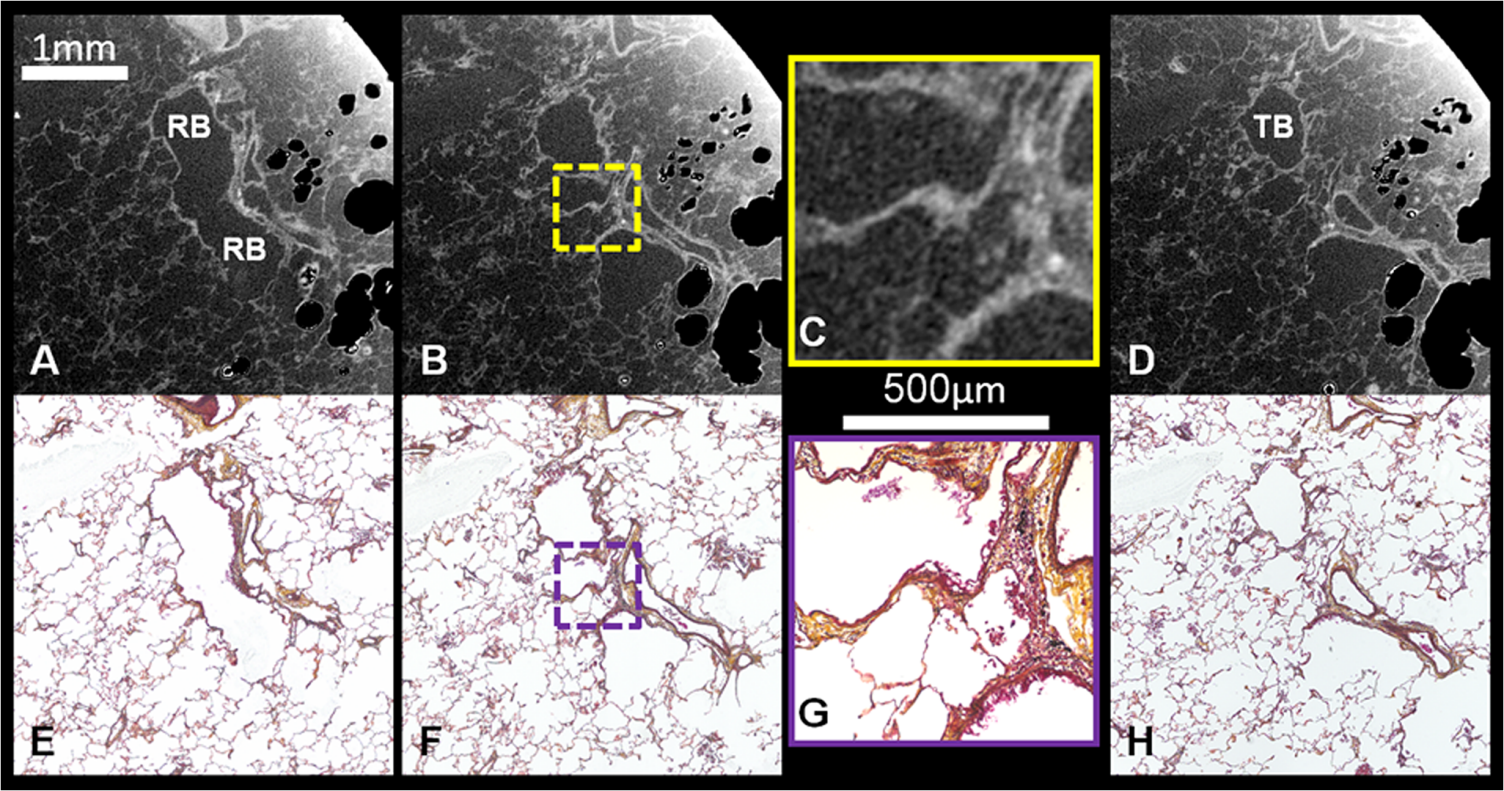
Matched serial μCT and Movat’s Pentachrome stained histological sections. Serial μCT images (A-D) and corresponding Movat’s stained histological sections (E-H) demonstrate the ability of the μCT scanner to provide sufficient contrast between tissue and paraffin to allow image analysis. Structures at the cellular level can be differentiated through staining of histological sections. Microscopy can achieve higher resolution as visualized by the magnified comparison of C (zoomed image) and G (20x magnification). [TB = terminal bronchiole, RB = respiratory bronchiole].

One advantage of obtaining μCT scans of FFPE samples is that the 3D information can be used to scout for regions of interest within a sample prior to sectioning for histology. As an example Fig 3 demonstrates matched serial μCT images and histological sections stained with Movat’s pentachrome stain. From the serial sections one can follow the terminal bronchiole into the respiratory bronchioles which are labelled correspondingly. Using this scouting technique, samples can be re-orientated and areas of interest can be identified without time intensive sectioning and staining of the entire sample. Further histological stains and light microscopy can be used to understand the tissue composition of the FFPE sample. As an example panel C shows a magnified area from the μCT section and panel G shows the corresponding histological section at a magnification of 20x. In this example Movat’s pentachrome stain is used to identify specific tissues such as collagen (yellow) and elastin fibers (black) which is not possible by μCT alone.

**Fig 4:**
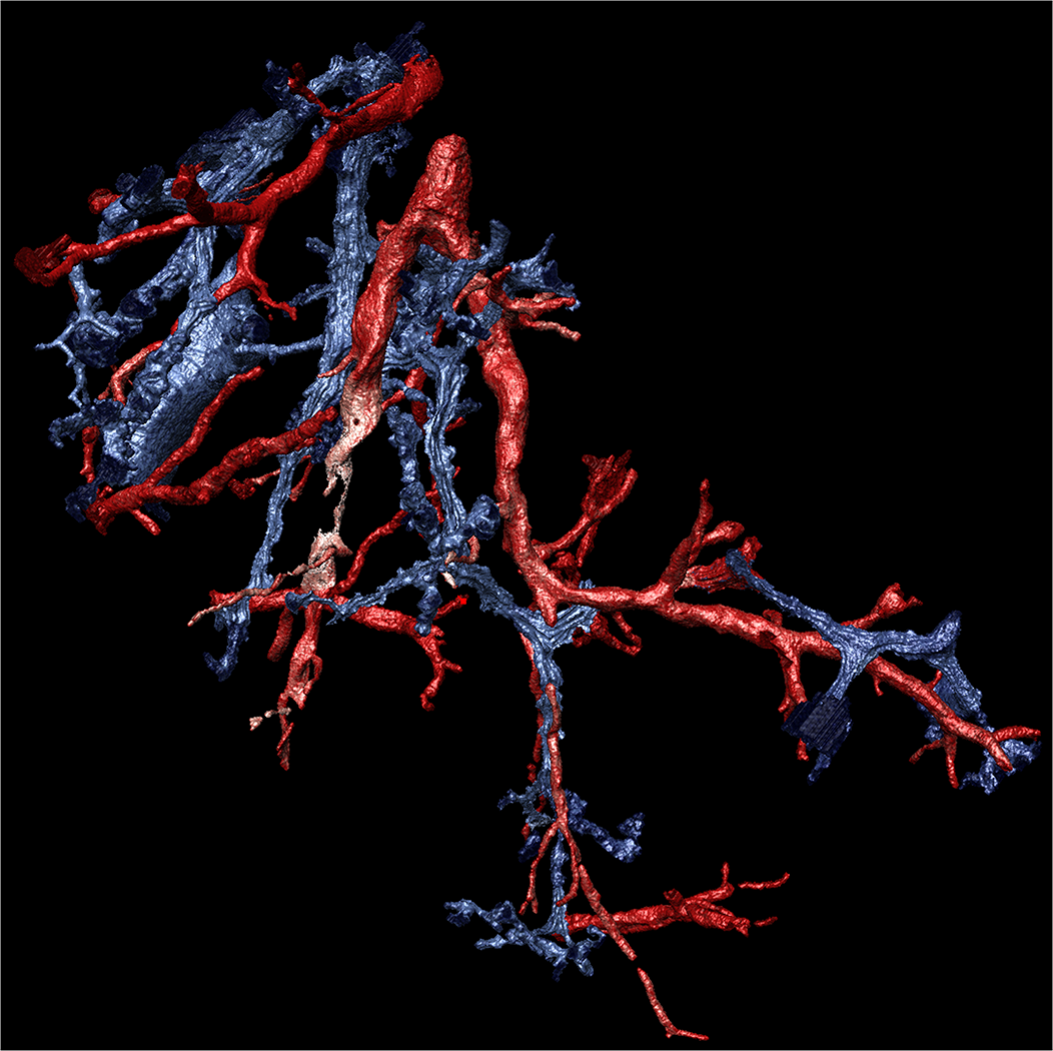
3D rendering of small airways and blood vessels. A 3D rendering of the segmentation of small airways (blue) and blood vessels (red) extracted from the μCT images of the FFPE sample also shown in Fig 2. Manual definition of the airway or vessel profile was combined with use of an automatic 3D seed growth tool (VGStudio Max) to delineate connected structures.

The μCT images of FFPE samples provide sufficient contrast to perform semi-automatic image segmentation of airways and blood vessels as shown as volume renderings in Fig 4. To ensure that structures were correctly identified, the small airways were traced in 3D to the first occurrence of alveolar openings. Such segmentations enable morphometric analysis, i.e. airway and vessel diameters, branch lengths and branching angles. The 3D rendering of segmentations allow the viewer to identify changes in the diameter and patency of airways along their length.

**Fig 5:**
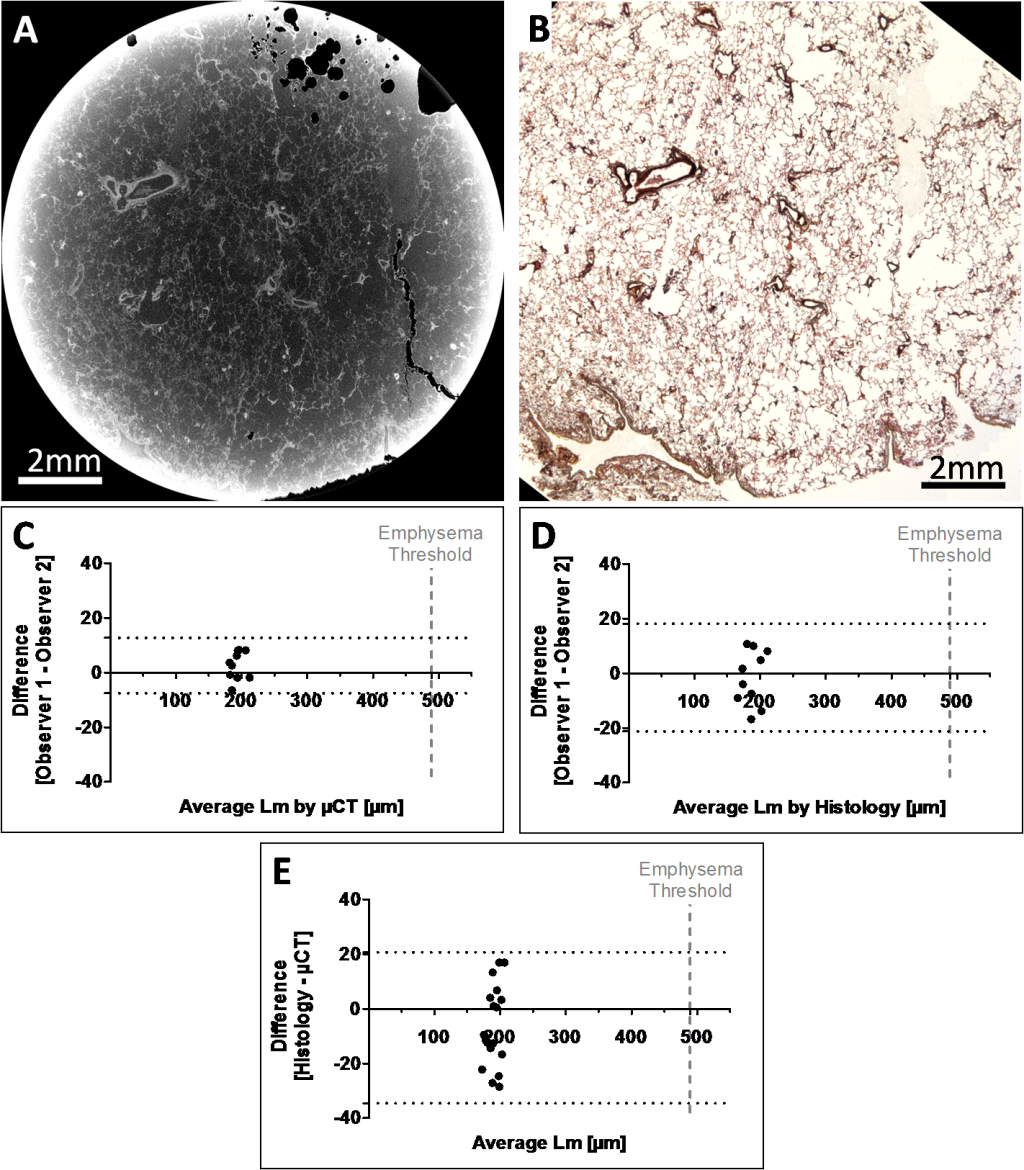
Comparison of mean linear intercept measurements. (A) Representative micro CT slice from a formalin fixed-paraffin embedded (FFPE) core and (B) the corresponding histology section at the same resolution (imaged with an objective of 2x magnification) matched using image registration. 10 μCT slices matched to 10 histological sections were randomly selected from one of the scanned samples and mean linear intercept counting was performed on all images. The Bland-Altman plots show the comparison of mean linear intercept counts between the 2 readers (co-authors TLH and DMV), for (C) micro CT, (D) histology, and (E) demonstrates the comparison of mean linear intercept measurements between the two imaging modalities.

Mean linear intercept (Lm) is generally used to assess alveolar air space size and can be used to determine if enlargement occurs with disease. In Fig 5 we measured Lm to determine if the resolution of the μCT scans was sufficient for such a measurement. To validate the Lm values we calculated Lm on 10 evenly distributed, μCT images and corresponding histological sections, over the height of the FFPE sample. To perform a direct comparison the magnification was matched between the imaging modalities and so images taken at a 2x magnification by light microscopy were compared to the μCT images. The Lm measurements conducted by the two independent readers are demonstrated in the Bland-Altman plots. Fig 5C and 5D demonstrate the non-significant inter-observer variability of Lm counted on μCT and histology images, respectively. The Bland-Altman plot in Fig 5E, shows the close correlation between the Lm measurements calculated from the matched μCT and histology images. However, it remains to be tested if μCT could be used to assess Lm in FFPE samples from patients with disease.

## Discussion

We demonstrate here that routinely prepared FFPE samples without the use of contrasting agents can be effectively imaged by laboratory μCT. Specifically we identified that there is a narrow X-ray attenuation contrast window between tissue and paraffin, which can be exploited for μCT imaging. To obtain optimal image quality to visualize the lung microstructure we describe a μCT protocol that requires a low flux and low kV setting, along with a reasonable scan time (order of hours). This imaging protocol allows multiple image-based analyses which include: 1) visualization of airways, vasculature and alveolar septae, 2) semi-automatic segmentation of airways and vessels to permit 3D rendering, 3) scouting for sites of interest within the tissue samples, and 4) measurement of lung structures. The benefits of μCT imaging include the acquisition of 3D datasets, enabling virtual sectioning in any orientation of the sample for a better understanding of the complex architecture of the lung. With two independent readers, there was also no significant variability between Lm measurements performed on matched μCT and histology images obtained from the FFPE samples. Additionally μCT imaging is non-destructive allowing scouting for regions of interest and subsequent use of the tissue for other techniques. Specific structures of interest can be sectioned or sampled in a more appropriate orientation for further, more comprehensive analysis at a higher magnifications by histological and immuno-histochemical staining.

In a previous study we have shown the advantages of laboratory μCT to dissect out the complexities of the distal airways disease in patients with end stage COPD [3]. In this study we highlighted the significant narrowing and obliteration of terminal bronchioles in severe COPD patients who were being treated with lung transplantation. However, the fixation technique we described included staining with osmium tetroxide and critical point drying, to provide significant contrast for μCT, a protocol which cannot be applied retroactively to archival FFPE samples. In contrast to the previous μCT studies described we demonstrate here that our scanning protocol of FFPE samples does not require additional staining or the use of contrast agents. The ability to image conventionally prepared FFPE samples is therefore a significant advance.

While there are several advantages to this technique, there are also multiple processing-induced artifacts that need to be considered. Formalin fixation is known to induce tissue shrinkage. However, it has been shown that the shrinkage is relatively homogenous and can therefore be corrected for [24]. As visualized by the μCT scans the paraffin infiltration of the large samples is not consistent throughout all samples, and in such cases can lead to microscopic air bubbles that prevent the visualization of thin septal walls in those areas. To achieve a resolution of 6.7μm, the width of the sample included in the field of view needs to be 15mm or less in diameter. As demonstrated by our μCT images if the sample is larger than the field of view, when the reconstruction algorithm is applied to the dataset it generates a bright halo artifact around the sample and an intensity gradient towards the center of the images. While these reconstruction based artifacts did not hinder our analysis because we excluded these regions from our analysis and focused only on the central regions of the FFPE samples, this could be improved by imaging smaller samples and/or use of more advanced reconstruction methods. We also acknowledge that images of FFPE samples have a much lower contrast than air-inflated, fixed and dried tissue samples. However, the clear advantages presented in our assessment protocol outweigh the disadvantage of reduced contrast.

An on-going μCT imaging challenge is to optimize the workflow to balance scan settings, duration and post-processing methodology for accuracy, reproducibility and operator work load. Furthermore, advanced image processing algorithms have to be developed in the future to expand our analysis of the 3D datasets. The potential to automatically segment and analyze the 3D morphometry of airways and vessels will provide a more comprehensive understanding of lung anatomy and help us to quantify remodeling that occurs in lung diseases such as asthma and COPD. FFPE samples obtained following a systematic uniform random sampling procedure can then be used to fully characterize the entire lung by applying stereological counting of volume fractions, surface areas and numbers [10,25,26].

## Conclusion

We have demonstrated that it is now possible to visualize the lung microstructure in standard FFPE human lung tissue using a laboratory μCT scanner without the added use of contrast agents.
